# Rescue of acute ST-elevation myocardial infarction secondary to traumatic coronary artery rupture: a case report

**DOI:** 10.1093/ehjcr/ytaf402

**Published:** 2025-08-16

**Authors:** Guangfeng Sun, Shan Huang, Huiyuan Kang, Bin Wang

**Affiliations:** Department of Emergency, Xiamen Cardiovascular Hospital, Xiamen University, No. 2999 Jinshan Road, Huli District, Xiamen, China 361000; Department of Intensive Care Unit, Xiamen Cardiovascular Hospital, Xiamen University, No. 2999 Jinshan Road, Huli District, Xiamen, China 361000; Department of Emergency, Xiamen Cardiovascular Hospital, Xiamen University, No. 2999 Jinshan Road, Huli District, Xiamen, China 361000; Department of Emergency, Xiamen Cardiovascular Hospital, Xiamen University, No. 2999 Jinshan Road, Huli District, Xiamen, China 361000

**Keywords:** Acute myocardial infarction, Traumatic coronary artery rupture, Cardiac tamponade, Coronary artery bypass grafting, Extracorporeal membrane oxygenation, Case report

## Abstract

**Background:**

Trauma-related acute myocardial infarction represents a complex and high-risk condition in the emergency department, necessitating a range of sophisticated treatment strategies. Failure to provide timely and accurate intervention significantly increases the risk of short-term mortality.

**Case summary:**

We present the case of a 36-year-old male who was admitted to local hospital following a penetrating chest trauma. Relevant diagnostic evaluations revealed haemopneumothorax and shock. Following initial stabilization measures, the patient was urgently transferred to our institution due to haemopericardium, which was initially suspected to be secondary to traumatic aortic dissection. However, the admission electrocardiogram clearly demonstrated concurrent anterior and high lateral wall myocardial infarction. Coronary angiography revealed occlusion of the left anterior descending (LAD) artery. We attempted interventional therapy on the LAD to restore blood flow. However, intraoperative findings revealed complete rupture of the LAD. The patient was transferred to the cardiac surgery department for emergency coronary artery bypass grafting (CABG). The foreign body was successfully retrieved, and the CABG was completed without complications. The patient gradually recovered under short-term ECMO support and was discharged successfully. Follow-up assessments indicate a favourable recovery.

**Discussion:**

The mechanism underlying traumatic myocardial infarction is complex and variable. Given that emergency physicians and cardiologists often lack extensive experience in such cases, they should exercise caution, conduct a thorough assessment, and meticulously plan each step of diagnosis and treatment.

Learning points

- Coronary artery rupture can concurrently result in myocardial ischaemia and cardiac tamponade, as the vascular stump may spontaneously occlude following the onset for reasons that remain unclear.
- In patients with trauma-induced myocardial infarction, manipulating the guidewire and confirming its position during percutaneous coronary intervention is challenging. Considering both subintimal and extraluminal positioning is crucial for successful outcomes.

## Introduction

Trauma-induced myocardial infarction is a rare yet critical complication that can result in rapid patient mortality. Given the potential co-occurrence of haemorrhage, shock, infection, and fractures, the management of such cases presents significant challenges. We report a case of completely traumatic rupture of the left anterior descending (LAD) artery, resulting in myocardial infarction and cardiac tamponade. A multidisciplinary team successfully managed the patient through pericardiocentesis, identification of the aetiology, percutaneous coronary occlusion, surgical repair, and extracorporeal membrane oxygenation (ECMO) support. This comprehensive approach led to a favourable outcome. This case aims to raise awareness among emergency physicians and cardiologists about trauma-induced coronary artery rupture as a cause of myocardial infarction and to provide valuable guidance for managing similar cases.

## Summary figure

**Figure:**
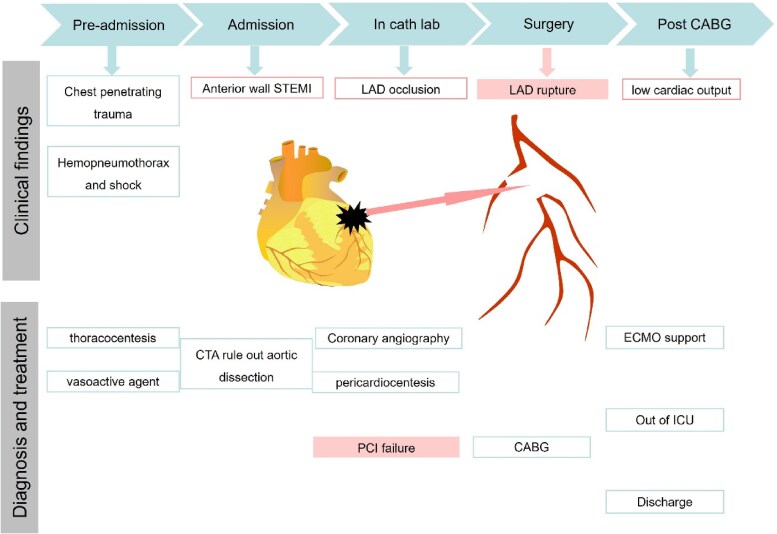


## Case presentation

**Figure 1:**
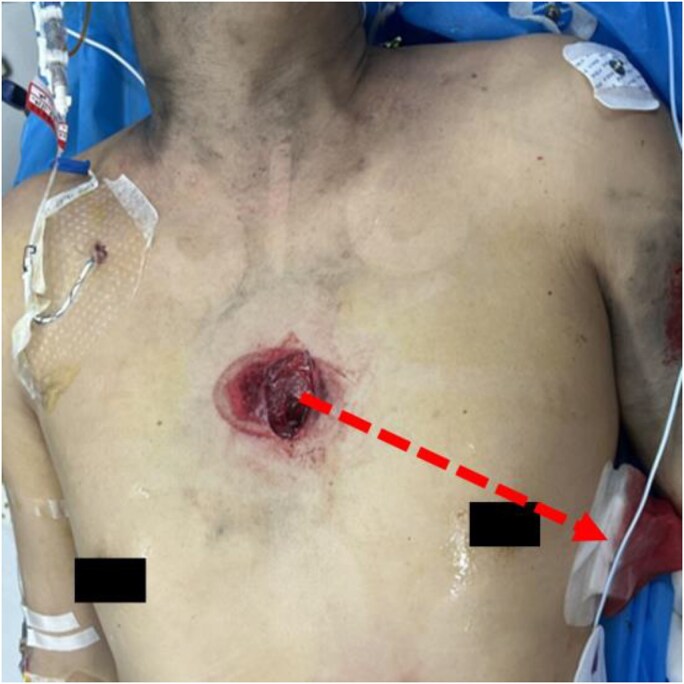
The entrance of the foreign material situated on the body surface and the hypothetical internal penetration path (dashed arrow).

A 36-year-old male was admitted to local hospital following a chest trauma. In view of the patient’s hypotensive state and the definitive diagnosis of left-sided haemopneumothorax, initial anti-shock measures were promptly initiated, followed by the implementation of left thoracic closed drainage. The patient was subsequently transferred to our centre for evaluation and management of significant haemopericardium. On arrival, physical examination indicated that a large screw penetrated the left thoracic cavity, with its tip protruding at the sixth intercostal space along the left midaxillary line (*Figure 1*). The drainage tube located on the left side of the thoracic cavity remains patent and effectively facilitates fluid removal.

**Figure 2:**
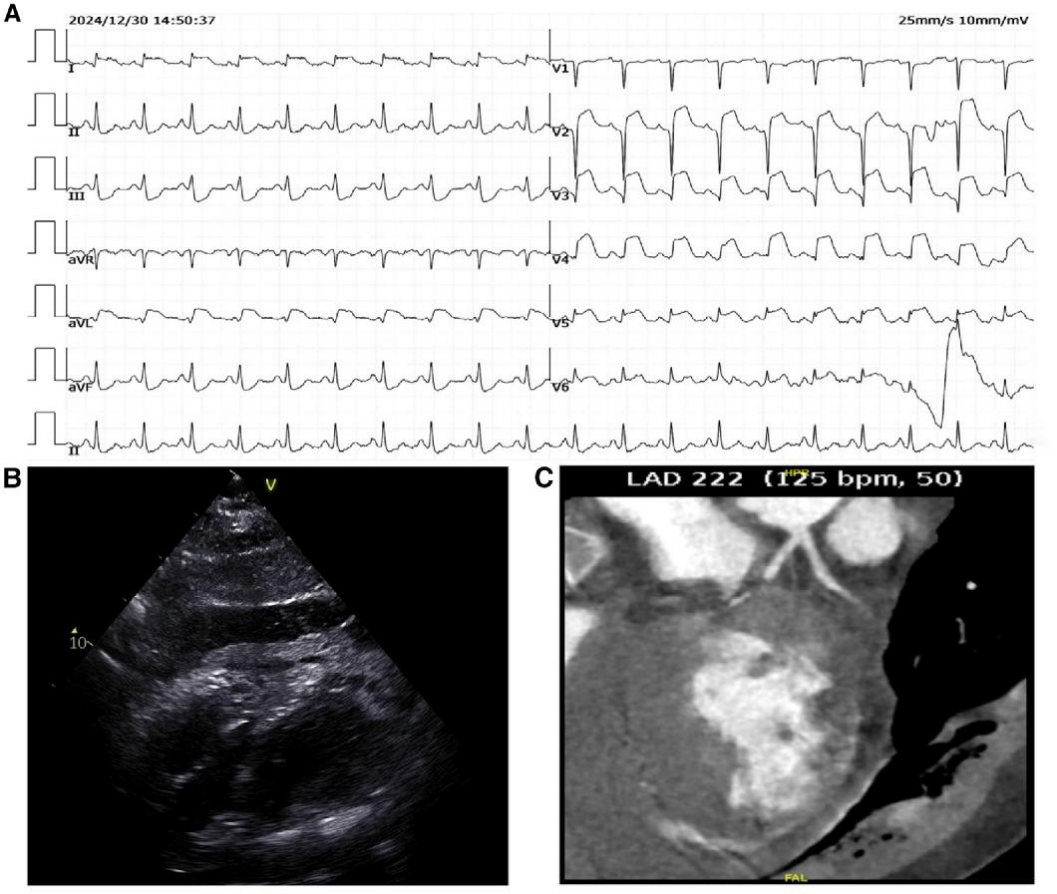
(*A*) The admission electrocardiogram showed characteristics of acute anterior and high lateral wall myocardial infarction. (*B*) The echocardiogram revealed a significant accumulation of pericardial effusion. (*C*) Coronary computed tomography angiography showed an absence of contrast agent opacification in the middle and distal segments of the left anterior descending artery.

The patient’s respiratory rate was recorded at 22 breaths per minute, with a blood pressure of 94/63 mm mercury (with dopamine 10 vg/min/kg) and a heart rate of 135 b.p.m. The electrocardiogram revealed a sinus tachycardia with a significant ST segment elevation in leads I, avL, and V2–V5 (*Figure 2A*). Cardiac troponin T rose to 751.71 pg/mL (normal reference limit 14 pg/mL). Echocardiography detected akinesis in the interventricular septum and mid-to-inferior left ventricle, with an left ventricular ejection fraction of 52%. Pericardial effusion was also found, mainly on the right ventricle’s free wall (14.2 mm) and atrioventricular groove (22 mm), with some areas forming blood clots (*Figure 2B*). To rule out aortic dissection as a common cause of myocardial infarction and pericardial effusion, we conducted computed tomography angiography imaging of the aorta and coronary arteries. Results showed no abnormalities in the thoracic and abdominal aorta. Notably, there was no contrast agent opacification in the mid-to-distal LAD and its diagonal branch (*Figure 2C*).

**Figure 3:**
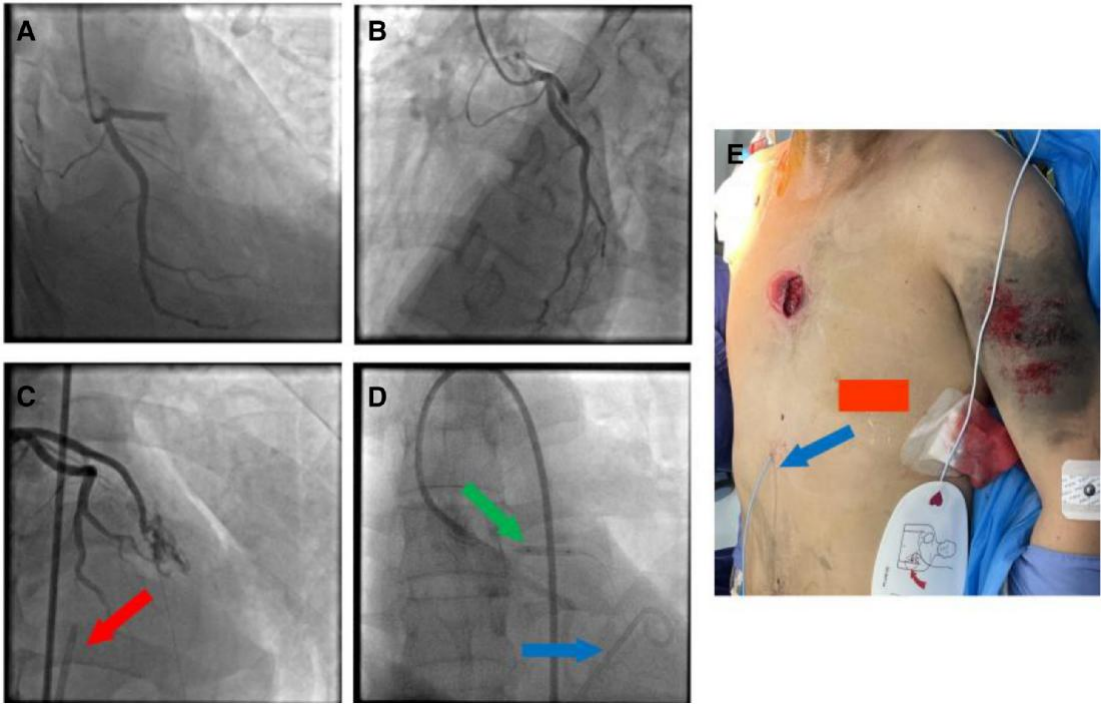
(*A* and *B*) Total occlusion of the proximal segment of left anterior descending artery. (*C*) Pericardiocentesis was performed after the coronary angiography (solid arrow), and when the guidewire was advanced through the occlusion segment, angiography showed a significant contrast extravasation. (*D* and *E*) A percutaneous transluminal coronary angioplasty balloon was continuously inflated in the proximal left anterior descending artery to closure the angiorhexis (green solid arrow in the middle), and a 5-F pigtail catheter was exchanged into the pericardial cavity to facilitate drainage (blue arrow).

Emergency coronary angiography verified complete occlusion of the proximal LAD artery (*Figure 3A* and *B*). Given the pericardial effusion at 22 mm in the atrioventricular groove, performing interventional procedures to restore LAD blood flow poses significant risks. Pericardiocentesis and drainage were performed before the coronary interventional procedure. After removing 500 mL of pericardial effusion, blood pressure stabilized. However, upon advancing the guidewire through the occluded segment of the LAD, its oscillation and subsequent angiography examination indicated an extraluminal position (see Supplementary data online, *Video S1*). Re-examination of angiography showed a significant contrast extravasation from the previously occluded site, suggesting complete rupture of LAD (*Figure 3C*). A Maverick^2^ 3.0 × 15 mm coronary angioplasty balloon (Boston Scientific Corporation, MA, USA) was continuously inflated in the proximal LAD to closure the angiorhexis (*Figure 3D* and *E*).

**Figure 4:**
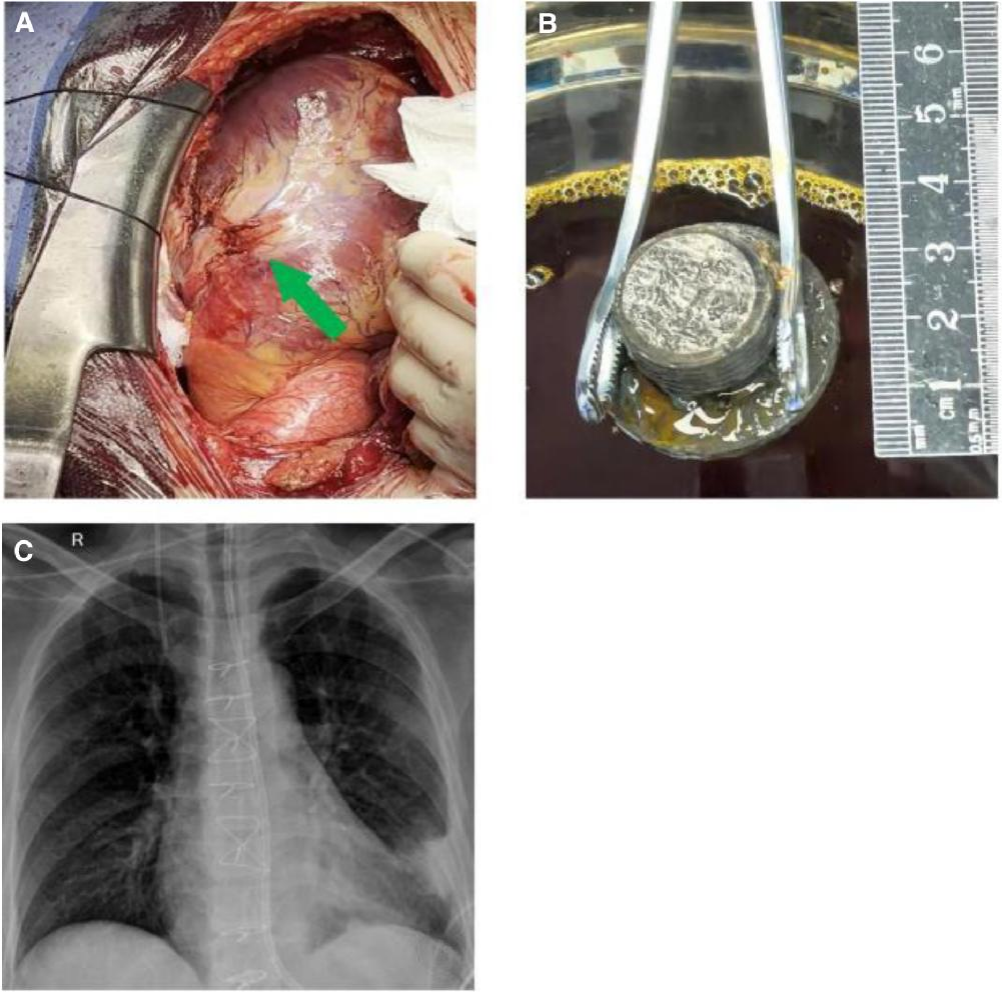
(*A*) The solid arrow shows a contusion of the left ventricular lateral wall and complete rupture of the left anterior descending artery. (*B*) The screw removed from the thoracic cavity during the surgery. (*C*) The post-surgery X-ray demonstrated an optimal recovery.

Due to the inability of the guidewire to enter the distal true lumen, we hypothesized that the patient’s LAD may have experienced complete rupture, with the distal segment retracting to an undetermined location. Given these conditions, further interventional attempts were deemed both futile and highly risky. Consequently, we promptly consulted the cardiothoracic surgical team for coronary artery bypass grafting. Intraoperative exploration revealed a contusion of the left ventricular lateral wall and complete rupture of the LAD (*Figure 4A*). The patient underwent off-pump aorta-saphenous vein-LAD bypass grafting with cardiopulmonary bypass on standby. A screw with a diameter of approximately 4 cm was identified on the left chest wall and successfully extracted (*Figure 4B*).

Given the patient’s post-operative low cardiac output and the administration of high-dose vasoactive agents, VA-ECMO was promptly initiated. This decision may be attributed to the absence of coronary artery disease history and lack of ischaemic preconditioning in the patient. After 6 days of VA-ECMO support in the intensive care unit (ICU), the patient’s cardiac function stabilized. The pump-controlled retrograde trial enabled successful ECMO discontinuation. Three days later, the endotracheal tube was removed. Two weeks into the ICU stay, a chest X-ray showed clear lung markings and significant improvement. The patient was transferred to a general ward and discharged on Day 37 without complications.

## Discussion

According to previous literature, approximately 5% of patients with thoracic injuries develop myocardial infarction.^1^ The coronary artery can be affected by various mechanisms, including dissection, sub-intimal haemorrhage, intraluminal thrombosis, spasm, or compression from an epicardial haematoma.^2–4^ Due to its anterior location directly over the heart, the LAD artery is the most susceptible coronary vessel to injury following trauma.^5^ There are about 30 case reports in PubMed linking trauma to myocardial infarction. The mechanisms include dissection in 13 patients, acute plaque rupture or thrombosis in 9, cardiac contusion in 1, and an artery-to-ventricle fistula in 1.^5,6^ Initially, we misdiagnosed this case as an intramural haematoma due to limited experience. The subsequent treatment contradicted our hypothesis, prompting us to revise and optimize our strategy. The following factors were derived from the successful outcome in this patient.

Firstly, patients with chest trauma often have distracting symptoms and a complex clinical picture, delaying accurate diagnosis and timely treatment of concurrent myocardial infarction.^7^ The mechanism of coronary artery occlusion in these patients is complex. We suspect that a thrombus forms after coronary artery rupture, as patients often have predisposing factors for thrombosis, including vascular spasm, subendothelial haematoma, and elevated inflammatory markers, which contribute to their vascular events.^4^ In our case, these mechanisms paradoxically became critical to the patient’s survival. This conclusion is based on current clinical theories and surgical findings, as large-scale clinical reports or autopsy data are lacking.

So secondly, pericardiocentesis must be conducted prior to the coronary interventional procedure to prevent a potential sudden exacerbation of tamponade. After the angiography confirmed the coronary artery rupture, we promptly replaced the conventional drainage tube with a 5-F pigtail catheter for optimal drainage. Simultaneously, a percutaneous transluminal coronary angioplasty balloon is essential for preventing ongoing haemorrhage. Upon encountering the inability to advance the guidewire into the distal vessel, we promptly opted for surgical intervention. Retrospectively, this decision proved critical in minimizing the patient’s total ischaemic time and was instrumental in saving their life.

Through this case, we highlight the critical need for clinicians to carefully differentiate the cause in patients with myocardial infarction following chest trauma. For interventional procedures, thorough assessment and comprehensive preparation for potential complications are essential.

## Lead author biography


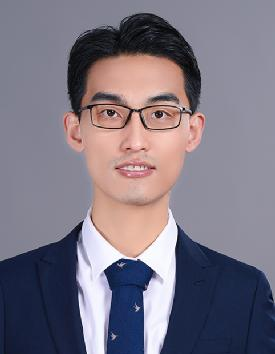


Guangfeng Sun, MD, is a cardiologist, and he is working at the Department of Emergency, Xiamen Cardiovascular Hospital, Xiamen University (Xiamen, China). He was born in 1993. His professional experience focuses mainly on critical heart disease, coronary intervention therapy, and ECMO implantation and management.

## Supplementary Material

ytaf402_Supplementary_Data

## Data Availability

The data underlying this article will be shared at reasonable request to the corresponding author.

## Notes

**Consent:** The authors confirm that they obtained the patient’s consent for submitting and publishing this case report in accordance with the COPE guidance.

**Funding:** This work was supported by Xiamen Science and Technology Project (Grant 2024GZL-QN134).
